# Biological Monitoring for Depleted Uranium Exposure in U.S. Veterans

**DOI:** 10.1289/ehp.0800413

**Published:** 2009-02-25

**Authors:** Carrie D. Dorsey, Susan M. Engelhardt, Katherine S. Squibb, Melissa A. McDiarmid

**Affiliations:** 1 Department of Medicine, University of Maryland School of Medicine, Baltimore, Maryland, USA; 2 Veterans Affairs Medical Center, Baltimore, Maryland, USA; 3 Department of Epidemiology and Preventive Medicine, University of Maryland School of Medicine, Baltimore, Maryland, USA

**Keywords:** bioassay, biomonitoring, depleted uranium, exposure, isotopic analysis

## Abstract

**Background:**

As part of an ongoing medical surveillance program for U.S. veterans exposed to depleted uranium (DU), biological monitoring of urine uranium (U) concentrations is offered to any veteran of the Gulf War and those serving in more recent conflicts (post-Gulf War veterans).

**Objectives:**

Since a previous report of surveillance findings in 2004, an improved methodology for determination of the isotopic ratio of U in urine (^235^U:^238^U) has been developed and allows for more definitive evaluation of DU exposure. This report updates previous findings.

**Methods:**

Veterans provide a 24-hr urine specimen and complete a DU exposure questionnaire. Specimens are sent to the Baltimore Veterans Affairs Medical Center for processing. Uranium concentration and isotopic ratio are measured using ICP-MS at the Armed Forces Institute of Pathology.

**Results:**

Between January 2003 and June 2008, we received 1,769 urine specimens for U analysis. The mean urine U measure was 0.009 μg U/g creatinine. Mean urine U concentrations for Gulf War and post-Gulf War veterans were 0.008 and 0.009 μg U/g creatinine, respectively. Only 3 of the 1,700 (0.01%) specimens for which we completed isotopic determination showed evidence of DU. Exposure histories confirmed that these three individuals had been involved in “friendly fire” incidents involving DU munitions or armored vehicles.

**Conclusions:**

No urine U measure with a “depleted” isotopic signature has been detected in U.S. veterans without a history of retained DU embedded fragments from previous injury. These findings suggest that future DU-related health harm is unlikely in veterans without DU fragments.

Responding to veterans’ concerns and as part of an ongoing medical surveillance program for U.S. military veterans exposed to depleted uranium (DU), biological monitoring of urine uranium (U) concentrations has been carried out by the Department of Veterans Affairs (VA) since the early 1990s. Since the first Gulf War in 1991, DU has been used both in armor-piercing projectiles and as armor itself, because of its high density, availability, and relative low cost [Army Environmental Policy Institute (AEPI) 1995].

A by-product of the U enrichment process, DU is the material remaining after the more radioactive U^234^ and U^235^ isotopes are removed from natural uranium (U_nat_) (AEPI 1995). DU thus possesses only about 60% of the radioactivity of U_nat_ but retains the elemental, chemical characteristics of this heavy metal.

Although DU was first deployed in the Gulf War in 1991 and was also used in the Bosnian conflicts, it has played less of a role in the present military campaigns in Iraq and Afghanistan. Despite this, health concerns persist about potential exposure risks, so surveillance efforts have been expanded to offer biomonitoring to any veteran requesting a test for DU exposure.

Veterans access the biomonitoring program through any VA clinic or hospital. The U.S. Navy and Air Force also use the VA’s DU biomonitoring program for their active-duty service members. The assessment includes a self-completed questionnaire and submission of a 24-hr urine sample, which is analyzed for total U concentration and for the presence of DU. With accompanying interpretation, results are returned to the veteran and his or her health care provider. The questionnaire addresses specific DU exposure scenarios of concern to veterans. Examples of potential exposure opportunities include inhalation of smoke containing DU particles (e.g., during a fire involving DU weapons at Doha depot, Qatar), entering or salvaging vehicles or bunkers hit by DU projectiles, and reporting close contact with DU munitions in tanks and other vehicles.

Results of surveillance completed between August 1998 and December 1999 and between January 2000 and December 2002 have been previously reported (McDiarmid et al. 2001a, 2004a). Both studies concluded that a veteran without a history of traumatic injury involving DU munitions would be unlikely to have an elevated urine U value.

In the 5 years since the last report of these surveillance results, 1,769 additional urine samples have been measured. In addition, we can now accurately perform determinations of U isotopic signatures at very low total U concentrations, enabling accurate identification of DU as opposed to U_nat_. This capability has significantly enhanced our ability to assess DU exposure. We report here an update of these results.

## Materials and Methods

### Program enrollment

From January 2003 through June 2008, 1,769 veterans and active-duty service members from the U.S. Navy and Air Force who served in the 1991 Gulf War and/or who have served since the end of the Gulf War (referred to as post-Gulf War veterans) provided 24-hr urine samples for U testing. The latter group includes veterans of the military conflicts in Iraq, Afghanistan, and the Balkans. The veterans’ local VA or the service members’ medical treatment facility contacted the DU program at the Baltimore VA Medical Center (BVAMC) to request a test kit and coordinated collection of the urine sample. As previously reported, the kit contained a demographic and exposure questionnaire, instructions for the collection and handling of the 24-hr urine specimen, and urine collection containers (McDiarmid et al. 2001a, 2004a). The collected specimen was returned to the BVAMC for processing. This protocol has been reviewed and approved by the University of Maryland School of Medicine Institutional Review Board and the Research and Development Office at the BVAMC. Personnel at the local VA hospital, clinic, or medical treatment facility obtained consent for medical evaluation. Informed consent requirements for the purposes of population surveillance were waived after review by the University of Maryland School of Medicine Institutional Review Board.

### Urine U analytic methods

Measurements of total volume and creatinine in the 24-hr urine samples were made at the BVAMC clinical labs. Before July 2003, U concentrations for Gulf War veterans were measured by STL Richland (formerly Quanterra, Inc., and International Technology Analytic Services; Richland, WA) using kinetic phosphorescence analysis (KPA). When urine U concentrations were > 0.05 μg U/g creatinine, patients were asked to submit a second sample for confirmation of the U concentration. Additionally, the presence of DU versus U_nat_ in the samples was determined by isotopic analysis at the Armed Forces Institute of Pathology (AFIP) in Washington, DC, using inductively coupled plasma mass spectrometry (ICP-MS) as described by Ejnik et al. (2005). Since July 2003, all urine samples, including those from Gulf War veterans, have undergone U quantification and isotopic analysis at AFIP.

### Urine U distribution

We dichotomized urine U values into high U (≥ 0.05 μg U/g creatinine) and low U (< 0.05 μg U/g creatinine) for statistical analysis. The 0.05 μg U/g creatinine cut-point has been used in prior publications related to this surveillance population (McDiarmid et al. 2001a, 2004a). We chose the value of 0.05 μg U/g creatinine because it was the approximate upper limit of the urine U distribution for nonoccupationally exposed, geographically divergent populations available when this project began in the mid-1990s (Dang et al. 1992; Medley et al. 1994). Although levels for the general population are now available through the National Health and Nutrition Examination Survey (NHANES) database [Centers for Disease Control and Prevention (CDC) 2005], the cut-point of 0.05 μg U/g creatinine has been retained to allow comparison with prior studies. Also, the cut-point is near the NHANES 95th percentile level (0.043 μg/g creatinine) for the general U.S. population ≥ 20 years of age (CDC 2005).

### DU exposure history assessment

As described previously (McDiarmid et al. 2001a), a team of VA and Department of Defense occupational health experts, health physicists, and field experts crafted a DU exposure questionnaire for use by military personnel. In the past, relationships between urine U concentration and exposure history were evaluated for the entire surveillance population. These analyses revealed that the presence of a retained DU fragment from a traumatic injury was the only predictor of an elevated urine U concentration. For this study, we analyzed exposure from questionnaire responses for only the subset of samples with a urine U concentration ≥ 0.05 μg U/g creatinine or isotopic analysis consistent with DU (*n* = 30).

## Results

**Figure 1:**
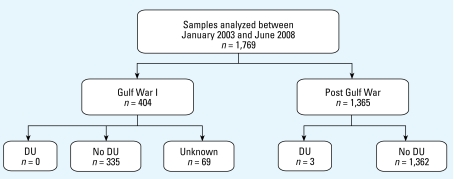
Summary of urine U surveillance activities from January 2003 through June 2008. The number of samples analyzed is stratified into Gulf War and post-Gulf War veterans. The results of isotopic analyses, which define the presence of DU are presented. “Unknown” indicates samples obtained before July 2003, when routine isotopic analysis on samples was initiated. The three samples with an isotopic signature consistent with DU were from individuals identified to have been injured as a result of friendly fire.

Between January 2003 and June 2008, we received 1,769 urine specimens for U analysis: 404 specimens from veterans of the Gulf War and 1,365 from post-Gulf War veterans. Figure 1 provides a summary of the urine U biomonitoring activity and the isotopic findings.

### Urine U measures

Table 1 summarizes the mean and median urine U measures. The mean urine U concentration for the entire population of 1,769 individuals was 0.009 μg U/g creatinine. Mean urine U concentrations for Gulf War and post-Gulf War veterans were 0.008 and 0.009 μg U/g creatinine, respectively, and did not differ statistically (*p* = 0.869). The median urine U measures were 0.004 (total group), 0.005 (Gulf War), and 0.004 (post-Gulf War) μg U/g creatinine.

Isotopic analysis was performed on 1,700 of the 1,769 (96%) specimens. The presence or absence of DU is determined by the ^235^U:^238^U ratio. A ratio between 0.0020 and 0.0030 defines a sample that is primarily DU. A ratio between 0.006 and 0.009 defines U_nat_. A ratio between these two ranges describes a sample that is a mixture of DU and U_nat_. Only 3 of the 1,700 (0.01%) specimens had an isotopic signature consistent with DU (ratios = 0.0029, 0.0029, and 0.0030). Mean urine U levels are higher in the individuals with DU compared with those with no DU detected in the urine (0.036 vs. 0.008 μg U/g creatinine). These individuals had been involved in friendly fire incidents and had retained DU fragments. As previous work has demonstrated, veterans with retained DU fragments continue to have elevated urine U levels compared with those without embedded fragments (McDiarmid et al. 2000, 2001b, 2004b, 2006, 2007, 2009). The remaining 1,697 specimens contained U_nat_ at levels that would be expected in the general U.S. population.

**Figure 2:**
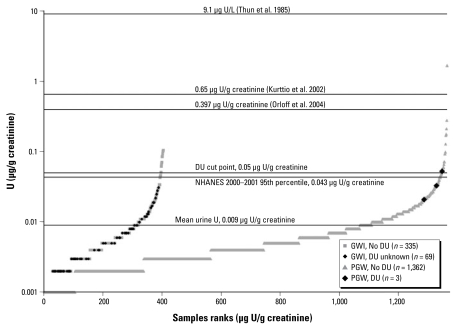
Distribution of urine U results from mail-in specimens from January 2003 through June 2008 plotted as two separate groups: Gulf War (GWI) versus post-Gulf War (PGW) veterans. Samples are ranked from low to high urine U concentration. The different symbols within each group denote whether DU was identified in each of the urine samples. Only three specimens were isotopically consistent with DU in the PGW group. Reference lines are provided on this graph for comparison purposes: Mean total urine U found in a subcohort of U fabrication workers in 1980 (Thun et al. 1985); Means from two populations with known high levels of environmental exposure to U_nat_ (Kurttio et al. 2002; Orloff et al. 2004); Cut-point established by the Depleted Uranium Follow-up Program to identify high versus low urine U concentrations (McDiarmid et al. 2001a); 95th percentile for urine U from the NHANES 2000–2001 population study for adults ≥ 20 years of age (CDC 2005); mean from the current cohort of 1,769 reported in the present study.

Figure 2 shows the distribution of urine U values ranked from low to high urine U (μg U/g creatinine) for both the Gulf War and post-Gulf War veterans. The urine U values ranged from < 0.001 to 0.105 μg U/g creatinine for the Gulf War specimens and from < 0.001 to 1.686 μg U/g creatinine for post-Gulf War specimens. The three specimens isotopically identified as having DU are indicated in Figure 2. Stratifying results into low (< 0.05 μg U/g creatinine) and high (≥ 0.05 μg U/g creatinine) urine U groups resulted in only 2.2% of Gulf War samples and 1.5% of specimens from post-Gulf War veterans being in the “high” range. Horizontal lines are shown for comparing the urine U concentrations obtained from the mail-in biomonitoring program with urine U concentrations that occur from environmental and occupational exposures.

Ninety-seven percent (97%) of Gulf War and 98% of post-Gulf War veterans had urine U values below the 95th percentile value of 0.043 μg U/g creatinine for the general U.S. population ≥ 20 years of age (CDC 2005). In addition, as shown in Figure 2, most sample concentrations fell below the mean urine U values found in two populations exposed to high U_nat_ concentrations in drinking water supplies: 0.65 μg U/g creatinine (Kurttio et al. 2002) and 0.397 μg U/g creatinine (Orloff et al. 2004). Nearly all urine U measurements were at least an order of magnitude below occupational exposures reported for U fabrication workers in 1980 (Thun et al. 1985).

### Exposure scenarios predictive of DU presence in urine

Thirty urine samples had a U concentration ≥ 0.05 μg U/g creatinine and/or were isotopically consistent with DU. Review of the questionnaires submitted by these veterans revealed that only the presence of a retained fragment and involvement in an incident known to involve DU munitions or DU armored vehicles predicted the presence of DU in the urine. This observation is consistent with previous findings from mailed-in specimens sent before January 2003 (McDiarmid et al. 2001a, 2004a).

## Discussion

The overwhelming majority (97%) of the 1,769 urine specimens submitted by Gulf War and post-Gulf War veterans had urine U levels in the range of those found in the general U.S. population, that is, ≤95th percentile of NHANES results collected between 2001 and 2002 (CDC 2005). More importantly, only 3 of the 1,700 urine specimens that underwent isotopic analysis had a DU isotopic signature. These three came from individuals reporting embedded fragments from DU friendly fire–related injury.

Only a small percentage of samples (2.2% Gulf War and 1.5% post-Gulf War) had urine U concentrations above the DU surveillance program’s cut-point of 0.05 μg U/g creatinine. The vast majority of these samples were U_nat_. The source of exposure for these individuals was most likely drinking water. The concentration of U in drinking water varies across the United States and the world, depending on the composition of the local bedrock. For example, unusually high concentrations of U_nat_ in drinking water have been found in areas of Finland (Kurttio et al. 2002), Canada (Zamora et al. 1998), and the United States (Orloff et al. 2004). Another potential source of U_nat_ exposure may be related to residence near a uranium mine or mill.

The findings of this study are generally consistent with those reported previously for surveillance of Gulf War personnel performed between August 1998 and December 1999 (McDiarmid 2001a) and from January 2000 through December 2002 (McDiarmid 2004a) (see Table 1). We found no statistical difference in the means of the urine U between the 169 samples received from 1998 through 1999 and the 277 samples received between 2000 and 2002 (the earlier reports of mail-in surveillance results cited above). However, when we compared mean urine U measures for these two cohorts (0.020 and 0.023 μg U/g creatinine) with the mean of the 1,769 samples reported here (0.009 μg U/g creatinine), we observed a statistically significant difference (*p* = 0.001). This difference can be explained by the change in the methodology used to measure the urine U concentration value. As noted above, starting in July 2003 samples were analyzed by ICP-MS. This methodology is more sensitive at lower urine U concentrations than is the KPA method formerly used to measure urine U concentrations. For samples collected and analyzed before 2003, we used the detection limit value for the KPA method in the calculation of the creatinine-standardized urine U concentrations for samples with U concentrations at or below the detection limit. This small overestimate of urine U concentrations is likely the basis for the higher group averages calculated for the earlier two cohorts.

As mentioned above, isotopic analysis of the samples examined for this study detected only three individuals who are excreting DU in their urine. Levels of urine U are higher in this group of three compared with the isotopically U_nat_ group (0.037 vs. 0.009 μg U/g creatinine). When we consider the entire surveillance population, dating back to 1998 (*n*= 2,246), only a total of four individuals have been identified as excreting DU in their urine. Exposure history confirms that these four individuals were injured as a result of friendly fire and have retained embedded fragments.

The results reported here are also consistent with those of another Department of Veterans Affairs–sponsored DU surveillance program, which has been following for 15 years a dynamic cohort of 77 Gulf War veterans with known exposure to DU from documented friendly fire incidents during the 1991 Gulf War. Among this group, only those with retained DU fragments from traumatic injury continue to excrete higher concentrations of U in their urine (McDiarmid et al. 2000, 2001b, 2004b, 2006, 2007, 2009). Urine U levels in those without retained DU fragments, but who sustained a historically documented inhalation exposure during the friendly fire incidents, are similar to general population levels and are isotopically consistent with U_nat_.

The persistent elevation of urine U observed in those with retained fragments is supported by animal studies in which DU pellets implanted into the animals (Hahn et al. 2002; Pellmar et al. 1999) oxidized *in situ*, thus serving as a metal depot for ongoing systemic exposure. As the metal ions are released to the circulation, they are filtered by the kidney and excreted in urine, resulting in higher U concentrations.

The data reported here on this expanded surveillance cohort of both Gulf War and more recently deployed soldiers and veterans confirm previous findings that, in the absence of retained DU fragments, it is highly unlikely that an individual will have chronically elevated urine U.

Historically, concern has been raised about the long lag time between exposure during deployment and subsequent biomonitoring performed years later. A transient U elevation from a one-time inhalation exposure could be missed if sampling occurred too long after U exposure, whereas measurements taken at the time of a potential exposure incident may indicate that an exposure to DU has occurred. Estimates reported by the Royal Society of London (2002) suggest that a DU oxide inhalation dose sufficiently high to cause significant health effects would still be detectable 10 years after exposure, using analytical techniques currently available.

The rate of elimination of an inhaled dose of DU depends on many parameters, including the chemical and physical form of the DU oxide particles inhaled and the exposure dose. Data collected as part of a U.S. Army DU aerosol characterization and risk assessment study, which characterized DU aerosols created by the perforation of an Abrams tank and a Bradley Fighting Vehicle with a large-caliber DU penetrator, indicate that soldiers in friendly fire incidents inhaled DU that was a mixture of soluble and insoluble DU oxides (Parkhurst et al. 2005). Soluble oxides would have been rapidly absorbed through the lungs and cleared by the kidney. Insoluble oxides would have been phagocytized by lung macrophages and cleared from alveolar areas either through the mucocilliary system to the mouth or by transport to lung-associated lymph nodes.

We also address the concern regarding the lag time between exposure and biomonitoring by including veterans from recent military conflicts in our surveillance program. We achieved a shortened time interval between potential initial exposure and assessment of urine U with this group, because we obtained many of these specimens within several weeks of the veterans’ return from deployment. Closing this gap between potential exposure opportunity and sample collection and the fact that we are still observing normal urine U results gives some reassurance that large numbers of soldiers from previous conflicts likely did not incur urine U elevations that were missed because of the delay in sampling.

Even if participants in the Gulf War conflicts had transient exposure to DU, it is highly unlikely that this would result in significant, subsequent health effects for several reasons. The clearance of U from the body is relatively rapid (nearly two-thirds of an acute dose is cleared through the kidney in 24 hr; Agency for Toxic Substances and Disease Registry 1999). Acute effects of U on the kidney (the primary target organ for soluble U compounds) do not persist after short exposures, and only under chronic exposure conditions does accumulation of U in the kidney cause long-term effects (Royal Society of London 2002). In addition, the absence of expected effects is supported by findings from the DU Surveillance Program for friendly fire victims. In 15 years of follow-up, no clinically significant U-related health effects have been observed in the cohort, including those with retained DU fragments (McDiarmid et al. 2000, 2001b, 2004b, 2006, 2007, 2009), except for subtle changes in renal proximal tubule markers in veterans with retained fragments (McDiarmid et al. 2009).

To summarize, the U biomonitoring results obtained over three reporting periods—the present study and two previous reports (McDiarmid et al. 2001a; 2004a)—have shown that > 95% of the 2,246 total urine specimens collected since 1998 had urine U concentrations similar to those found in the general U.S. population (CDC 2005), and all but one sample, which has a U_nat_ isotopic signature, had U concentrations < the occupational decision level (0.8 μg/L) used by the Fernald Environmental Management Project for U-exposed workers (Fernauld Environmental Management Project 1997). In addition, review of exposure histories confirms previous findings that the presence of a retained DU fragment and history of friendly fire exposure are the best predictors of an elevated urine U concentration.

This indicates that for most veterans who are concerned about exposure to DU as a result of their deployment, urine U concentrations outside the normal range are a rare occurrence and DU isotopic signatures are even more uncommon. No persistent urine U elevations have been detected in potentially exposed veterans without a history of DU-embedded fragments. This further suggests that future DU-related health harm is unlikely.

## Figures and Tables

**Table 1:** Mean and median urine U values (μg U/g creatinine) in U.S. veterans.

	No.	Mean ± SE	Median
McDiarmid et al. (2001a)	169	0.020 ± 0.003	0.010
McDiarmid et al. (2004)	446^a^	0.023 ± 0.006	0.010
Samples from Gulf War veterans since January 2003	404	0.008 ± 0.001	0.005
Samples from post-Gulf War veterans^b^ since January 2003	1,365	0.009 ± 0.001	0.004
All samples since January 2003	1,769	0.009 ± 0.001	0.004

a Includes 169 samples from McDiarmid et al. (2001a).

b Veterans who have served since the first Gulf War, including those serving in Iraq, Afghanistan, and the Balkans.
